# 2019 National Health Survey: progress in monitoring the health of the Brazilian people

**DOI:** 10.1590/SS2237-9622202200001.especial

**Published:** 2022-08-08

**Authors:** Fatima Sonally Sousa Gondim, Maryane Oliveira Campos, Thaynã Ramos Flores, Giovanny Vinícius Araújo de França, Arnaldo Correa de Medeiros

**Affiliations:** 1 Ministério da Saúde, Secretaria de Vigilância em Saúde, Brasília, DF, Brazil

The National Health Survey (*Pesquisa Nacional de Saúde* - PNS) came into being with the purpose of expanding the thematic scope of the Health Supplements of the National Household Sample Survey (*Pesquisa Nacional por Amostra de Domicílios* - PNAD). The PNS was designed to collect information about the health conditions of the population, surveillance of chronic noncommunicable diseases (NCDs) and their associated risk factors, as well as to verify the performance of the national health system regarding access to and use of available services and continuity of care. The PNS was conducted for the first time in 2013, as a result of a formal agreement between the Ministry of Health and the Brazilian Institute of Geography and Statistics (IBGE).

In this second edition, conducted in 2019, the PNS presents greater maturity regarding methodological aspects, including extensive revision of the questionnaire. The 2019 PNS included new questions related to working conditions and fatherhood during prenatal care, for example. Among the great differentials of the 2019 PNS, for the first time, a household survey included, on a national scale, a module of questions intended to contribute to evaluating the quality of Primary Health Care (PHC) services. The survey also addressed the performance of preventive examinations, involvement with types of violence and, in addition, collected anthropometric data from one of the residents of each household visited.

Monitoring the health conditions of the population, through surveys such as the PNS, is essential for producing relevant information for informing assertive decision-making in the field of public health, contributing to building and evaluating health policies. As such, the Health Ministry’s Health Surveillance Secretariat (SVS/MS), together with partners such as the IBGE, reinforces its commitment by undertaking surveys that enable the health profile of the Brazilian people to be monitored, producing indicators necessary for monitoring the fulfillment of nationally proposed targets, as well as the commitments taken on by Brazil regarding strategic international agendas.

Drawing attention among the results of the 2019 PNS is the high prevalence of NCDs found among Brazilians. In 2019, 23.9% of adults interviewed reported having hypertension; 7.7%, diabetes *mellitus*; and 14.6%, high cholesterol. Brazil has achieved only two of the nine proposed goals for addressing chronic diseases,^1^ pointing to the need to expand and promotion of strategic actions aimed at addressing these diseases and their risk factors, given the ongoing aging of the Brazilian population.

Evaluation of the quality of PHC services within the Brazilian National Health System (*Sistema Único de Saúde* - SUS), from the perception of service users, was an innovation in the second edition of the PNS. Evaluation was achieved through the use of an internationally validated instrument, the Primary Care Assessment Tool (PCATool Brasil). Most Brazilians with hypertension (66.1%) reported being cared for in public health services, 45.8% of them in primary health care centers.^2^ In addition, regarding health promotion measures, most individuals with hypertension reported having received guidance from health professionals on management of the disease.^2^

Another important challenge for the SUS, highlighted in this Special Issue of journal Epidemiology and Health Services are health inequities. Analysis of hypertension and diabetes *mellitus* prevalence in individuals aged 60 years or older showed that those with less education were the most affected by both diseases.^3^ A study on the People’s Pharmacy (*Farmácia Popular*) showed that this Program still has important socioeconomic and regional inequalities in the provision of medication for hypertension and diabetes.^4^

Another alarming result of the second edition of the PNS was the increase in self-reported depression in Brazil, rising from 7.6%, in 2013, to 10.2% in 2019.^5^ The survey was innovative in its investigation of quality of sleep which, in turn, can contribute to the increase in cardiovascular, metabolic, respiratory and mental illnesses, among others. Regarding this matter, sleep problems self-reported by Brazilian adults were found to be associated with all diseases and multiple diseases assessed.^6^

In addition to the results highlighted here, this Special Edition, also includes data on oral health conditions, chronic kidney disease, working conditions and prenatal care, NCD risk and protective behaviors, smoking cessation, inequities associated with physical activity, involvement in violence.

Finally, we emphasize that the health surveys sponsored by the SVS/MS are continually being improved in order to cover the main Brazilian public health problems, so as to allow the production of information that can inform strategic actions to face these problems. The national surveys, such as the 2019 PNS, the Chronic Disease Risk and Protective Factors Surveillance Telephone Survey (VIGITEL)^7^ and the National School Health Survey (PeNSE), enable monitoring and evaluation of the Strategic Action Plan for Addressing Chronic Non-communicable Diseases and Health Conditions in Brazil, 2021-2030. It is a strategic document, which serves as a guideline for the prevention of NCD risk factors and for the promotion of the population’s health in order to reduce health inequalities, in line with the Sustainable Development Goals 2030 Agenda. It must also be emphasized that the Ministry of Health has based itself on the National Health Surveillance Policy, a public State initiative, essential for the universal and cross-cutting improvement of the SUS, guiding the health care model in the territories.^8^
